# Enhanced Photolysis and Photocatalysis Assisted by Hydrodynamic Cavitation for the Removal of Methylene Blue Dye

**DOI:** 10.1021/acsomega.6c03407

**Published:** 2026-08-06

**Authors:** Margarita Navarrete, Javier Alejandro Rosas, Daniel Aguirre Aguirre, Rodrigo Mayen Mondragón, Jorge Luis Naude de la Llave

**Affiliations:** † Universidad Nacional Autónoma de México, Instituto de Ingeniería, UNITA, Vía de la Innovación 410, PIIT-Monterrey Autopista Monterrey-Aeropuerto km 10, Apodaca, 66629 Nuevo León, México; ‡ 7180 Universidad Nacional Autónoma de México, Facultad de Ingeniería, C. U. Coyoacán, 04510 Ciudad de México, México; § Universidad Nacional Autónoma de México, Instituto de Ciencias Aplicadas y Tecnología, UNITA, Vía de la Innovación 410, PIIT-Monterrey Autopista Monterrey-Aeropuerto km 10, Apodaca, 66629 Nuevo León, México; ∥ Universidad Nacional Autónoma de México, Facultad de Química, UNITA, Vía de la Innovación 410, PIIT-Monterrey Autopista Monterrey-Aeropuerto km 10, Apodaca, 66629 Nuevo León, México

## Abstract

This study investigates the synergistic integration of hydrodynamic cavitation (HC) with photolysis and photocatalysis to enhance the degradation of synthetic dyes in aqueous systems. Methylene blue (MB) is selected as a model pollutant, and tap water is used as the working fluid. HC and UVC photolysis are evaluated independently as control experiments, resulting in four treatment configurations: hydrodynamic vaporous cavitation, dynamic flow-UVC photolysis, HC-UVC photolysis, and HC-UVC-ZnO photocatalysis. In the last two treatments, air flow is introduced at the Venturi throat, generating an aerated turbulent flow. The proposed recirculating HC-UVC platform combines controlled gas injection with independent residence-time control, providing a well-defined experimental basis for comparative evaluation and future scale-up. Dye degradation is monitored by UV–vis spectroscopy and supported by measurements of physicochemical water quality parameters, dissolved oxygen, and residual photocatalyst detection. Apparent kinetics analysis and electrical energy per order (*E*_EO_) are used to quantitatively compare process performance. Strong synergistic effects are observed when HC is coupled with photonic and catalytic processes. In particular, HC coupled with photocatalysis achieved rapid degradation kinetics and the lowest *E*_EO_, reaching 98% MB removal within less than 1 h. Despite its superior performance, photoluminescence analysis revealed residual ZnO powder below the detection limits of electrochemical and absorption techniques, indicating a potential limitation associated with downstream photocatalyst recovery. In contrast, HC coupled with photolysis achieved comparable degradation efficiencies at longer processing times while avoiding the use of solid catalysts. These results demonstrate that optimal process selection should balance degradation efficiency, energy consumption, system complexity, and post-treatment requirements, highlighting HC-UVC photolysis as a robust alternative for practical water treatment applications where simplicity and long-term operability are prioritized.

## Graphical abstract

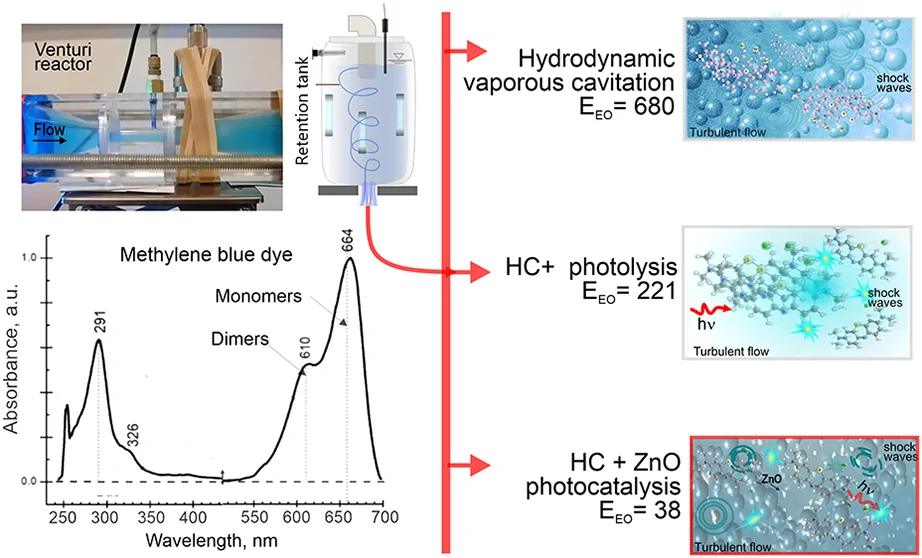

## 1 Introduction

Synthetic dyes represent a significant environmental concern due to their high stability, toxicity, and low biodegradability. Among industrial sectors, the textile industry is widely recognized as one of the largest sources of dye-contaminated effluents, generating large volumes during dyeing and finishing processes.^1–5^ Once discharged into aquatic environments, dyes can significantly reduce light penetration, inhibit photosynthetic activity, and pose risks to aquatic organisms and human health. Conventional methods for dye removal include adsorption and chemical oxidation.^4,6–10^ However, these approaches often suffer from limitations such as incomplete mineralization, secondary waste generation, and high operational costs. In this context, advanced oxidation processes (AOPs) have emerged as effective alternatives for the degradation of refractory organic pollutants in industrial wastewater.^9–12^ AOPs rely on the in situ generation of reactive oxygen species (ROS), particularly hydroxyl radicals (^•^OH), which react nonselectively with organic compounds at near diffusion-controlled rates.^13,14^ AOPs comprise a wide range of technologies, including photolysis and photocatalysis, Fenton and photo-Fenton processes, electrochemical oxidation, plasma-based techniques, ultrasound, hydrodynamic cavitation, and radiation-based methods.^9,12^ For example, wastewaters containing chlorophenols (CPs), which are highly toxic and poorly biodegradable, have been treated using AOPs.^47^ The degradation and mineralization of CPs by means of AOPs have been realized by techniques based on hydrogen peroxide (H_2_O_2_ + UV, Fenton, photo-Fenton, photolysis, and photocatalysis) and processes based on ozone (O_3_, O_3_ + UV, and O_3_ + catalyst). All of them are pathways in which different mechanisms are activated for degradation and mineralization.^18–21^ In addition, previous studies have extensively examined AOP not only in terms of degradation efficiency and energy consumption but also focusing on reaction mechanisms, degradation pathways, and the formation of intermediate byproducts during the oxidation of dyes such as methylene blue.^15^ These studies highlight that discoloration does not necessarily correspond to complete mineralization, as intermediate aromatic compounds and potentially toxic species may still be present in solution.^23–25^ For example, an adsorbing material may be efficient at trapping the MB monomer, but if it lacks a porous system to accommodate its clusters (such as dimers or trimers), it will be ineffective.^16,17^ Therefore, the electrical energy per order (*E*_EO_) value has been proposed as a meaningful figure of merit for comparing AOP performance and evaluating their probable scalability.^15^ Previous studies have reported that UV-photocatalysis and cavitation-driven processes often fall within the high-energy-consumption category, highlighting the need for intensified or hybrid strategies.

In this study, we investigate the degradation of methylene blue dissolved in tap water using a closed-loop hydraulic circuit equipped with a Venturi cavitation reactor and ultraviolet irradiation stages. Hydrodynamic cavitation is evaluated alone and in combination with photolysis and photocatalysis using ZnO as a model photocatalyst. The performance of each treatment configuration is quantitatively compared in terms of degradation efficiency, kinetic rate constants, synergistic indices, and water matrix composition, as well as practical considerations such as process simplicity and catalyst recovery. The results provide insights into the benefits and limitations of coupling hydrodynamic cavitation with photonic and catalytic AOPs for energy-efficient dye degradation in realistic water matrices. It is important to note that the results obtained in this study are based on UV–vis decolorization and surface characterization techniques, which provide evidence of dye removal but do not allow a definitive assessment of complete mineralization or the formation of byproducts.

The subsequent paragraphs provide an introduction to the physical chemistry involved in the applied processes; the reader may skip them if versed in the essentials of these processes.

### 1.1 Photolysis

It is a direct photodegradation process that uses ultraviolet (UV) or visible light to promote the transformation of molecules into smaller products; however, complete mineralization to CO_2_ and water cannot be assumed without specific analytical confirmation. When photons are absorbed by the dye molecules, exciting their chromophore groups, the excited species undergo reactions such as bond cleavage, isomerization, and decarboxylation, ultimately producing carbon dioxide (CO_2_) and water. This process is clean, as it does not produce sludge.^18–21^ Treating high concentrations of organic pollutants requires either increased energy input or higher H_2_O_2_ dosages. Most studies report removal efficiencies above 98% when using low H_2_O_2_/dye ratios. This is achieved either by extending the reaction time or by increasing the light power over 250 W.^3,9,10^ In some cases, high removal efficiency is reached with H_2_O_2_/dye ratios exceeding 2000 (in less than 15 min), which leads to reduced specific energy consumption.^16,22^

### 1.2 Photocatalysis

It is an indirect photolysis process that employs semiconductor materials such as zinc oxide (ZnO) or titanium dioxide (TiO_2_). When irradiated, the photocatalyst generates electron–hole pairs on its surface. These excited electrons react with oxygen molecules to form superoxide radicals (^•^O_2_^–^), while the holes produce hydroxyl radicals (^•^OH). These reactive species oxidize dye molecules and may promote further degradation.^23–25^ In the present study, however, complete mineralization was not directly confirmed.

### 1.3 Hydrodynamic Cavitation (HC)

has gained increasing attention as a highly energy-efficient process for tertiary-level water treatment.^26,27^ Orifice plates, Venturi tubes, constrictions, vortex cavitation devices, rotating hydrodynamic cavitation generators (RGHC), and microreactors have been successfully applied for the degradation of dyes such as MB, rhodamine B, methyl orange, that can achieve ^•^OH yields of ∼0.6 μg/mL after a few cycles-strongly outperforming macro-scale devices.^28–40^ Cavitation alone is recognized in the literature as a highly energy-efficiency process and is often considered suitable for commercial-scale implementation.^41–51^ Direct quantitative comparison with previous studies remains challenging in hydrodynamic cavitation systems due to the strong dependence on operating and geometric parameters—such as cavitation number, Reynolds number, reactor configuration, and residence time—that are often not consistently reported in the literature. Therefore, the present work focuses on maintaining controlled, well-defined operating conditions to enable consistent internal comparison across treatment configurations.

### 1.4 Water Matrix

The bulk composition of a water body includes major ions, dissolved organic carbon, suspended solids, redox-active species, and nutrients and other components. These constituents, commonly referred to as the water matrix, interact with both the target contaminants and the treatment processes, often modifying reaction rates. In particular, matrix components can influence hydroxyl radical chemistry and the reactivity of both dissolved organic and inorganic species.^13,14,52^ Even so, our tap water matrix was not characterized by a full compositional analysis. Instead, it was assessed through bulk physicochemical parameters relevant to process performance, including pH, total dissolved solids (TDS), electrical conductivity (EC), salt percentage, and temperature. Accordingly, the discussion of matrix effects is restricted to these bulk properties.

### 1.5 Chromophore Groups

Table 1 lists the chromophore group associated with each chemical substance used in this study, along with the corresponding electronic transition that occurs upon the absorption of UV–vis photons. Methylene blue (MB) is a basic cationic thiazine dye whosehigh stability and aromatic amine content make it resistant to degradation. For this reason, MB is often selected as a model compound in dye degradation studies.^13,14^ At doses greater than 5 mg/kg, the properties of MB dye as a monoamine oxidase inhibitor can induce fatal serotonin toxicity in humans, apart from being a threat to fauna in the aquatic ecosystem.^15,16^

**Table 1:** Chromophores Involved and their Electronic Transitions

Substance	Chromophore Group	Absorption [nm]	Molar Absorption [L·mol^–1^·cm–^1^]	Electronic Transition
Water	O–H	<190	10^–3^–10^–1^	s → s^a^
Methylene blue	Aromatic system + N	250–325	No data	Degradation products
		610–665		π → π^a^
			≈74,000	Charge-transfer
ZnO powder	Band gap	Bulk, 375	Depends on the surface^b^	Electron–hole pairs
		360–380		

a Excited state.

b Particle size (quantum effect), impurities or dopants, crystalline defects, and temperature and pressure.

### 1.6 Degradation Efficiency, Removal Kinetics, and Synergistic Index Calculations

To determine the degradation efficiency and the apparent pseudo-first-order kinetic parameters of MB removal, UV–vis spectrometry was employed.^18,27,33^ Absorbance values were used as a proxy for concentration, assuming proportionality according to the Beer–Lambert law. The maximum absorption peak of MB within the 520–695 nm range was selected for the analysis.

1 $$d\mathrm{egradation efficiency}\left(\%\right)=100\times \left(\frac{A_{0}-A_{t}}{A_{0}}\right)$$

where *A*_0_ is the initial absorbance, and *A*_*t*_ is the absorbance at time *t*.

Under the experimental conditions evaluated, the degradation kinetics were described using an apparent pseudo-first-order model, which accounts for the cumulative effects of reaction, mass transfer, and radical generation in the system. The apparent rate constant (*k*_app_, min^–1^) was obtained from

2 $$k_{\mathrm{app}}=-\frac{\ln\left(\frac{A_{t}}{A_{0}}\right)}{t}$$

The synergistic index (ξ) for a combined process is defined as

3 $$\xi =\frac{k_{\mathrm{comb}}}{k_{\mathrm{cav}}+k_{\mathrm{AOPs}}}$$

where *k*_comb_ is the apparent rate of the hybrid combined process, and *k*_cav_ and *k*_AOPs_ correspond to the individual processes.^49^

According to the recommendations of the Photochemistry Commission of the International Union of Pure and Applied Chemistry (IUPAC), for low pollutant concentrations -as in this case- the most appropriate figure of merit is the electrical energy per order (*E*_EO_), defined as the electrical energy required (kWh) to reduce the pollutant concentration by 1 order of magnitude (90%) in 1 m^3^ of water. The *E*_EO_ (kWhm^–3^ order^–1^) is calculated as

4 $$E_{\mathrm{EO}}=\frac{P\times t\times 1000}{V\times 60\times \log\left(\frac{C_{i}}{C_{f}}\right)}$$

where *P* is the lamp power (kW), *t* is the irradiation time (min), *V* is the treated volume (*L*), and *C*_i_ and *C*_f_ are the initial and final pollutant concentrations, respectively.^15,23,53^

### 1.7 Cavitation Number

Engineers use a parameter known as the cavitation number to assess the strength of cavitation, denoted as σ. This parameter is defined as σ = (p_d_–p_v_) (0.5ρ_L_V_th_^2^)^−1^ where *p*_v_ is the vaporous pressure of the saturated liquid at the corresponding temperature, *p*_d_ denotes the downstream pressure, ρ_L_ is the liquid density, and *V*_th_ indicates the mean throat liquid velocity. This parameter determines the extent of cavitation in hydrodynamic devices, disregarding thermodynamic effects. Separation zones also occur when the flow path geometry changes abruptly. Reaching a certain σ, periodic shedding of a bubble cloud can be established, which causes fluctuation in cavity length.^26,27,54–57^

## 2 Experimental Section

### 2.1 Chemicals and Equipment

Methylene blue (MB) powder dye with the formula C_16_N_3_H_18_SCl·3H_2_O (trihydrate compound) with a molecular weight of 373.91 g/mol was obtained from Herschi Trading. The aromatic fraction contains N and S atoms. It has dimethylamine groups attached to it. Its heteroaromatic fraction (phenothiazine) is planar, and the molecule is positively charged. Cosmetic-grade ZnO powder with CAS No. 1314–13–2 was obtained from IsaaQuim. Tap water from Apodaca (Nuevo León, México) had an average pH of 8.15 and conductivity of 781 μS cm^–1^ at 13 °C and a pH of 8.41 at 24 °C. Tap water contains traces of fluoride, chloride, and chloramine; minerals such as calcium, magnesium, sodium, and potassium; and trace elements such as iron, zinc, phosphorus, and manganese. The deionized water used had an average conductivity of 0.073 μS cm^–1^, a pH of 6.98 at 20 °C. For electrochemical analysis, we used a potentiostat (Bio-Logic SP-150) connected to three electrodes in a cylindrical cell containing the aqueous sample. A glassy carbon rod (3 mm in diameter) was used as working electrode (WE). A platinum wire (1.0 × 10 mm) served as the counter-electrode (CE). The reference electrode (RE) was mercurous sulfate in saturated potassium sulfate, with a standard potential of approximately +0.64 V relative to the standard hydrogen electrode (SHE). Spectrometric measurements were performed under ambient conditions over the wavelength range of 244 and 700 nm using high-resolution Ocean Optics HR2000+ spectrometer, model HR + C1513. The UVC lamps used are commercial grade for domestic and aquatic purposes. We used three 25 W external ozone lamps, three 13 W submersible lamps, and one 22 W submersible lamp. Figure 1 shows the emission spectra of the UVC lamps used in this study. The lamps have standard quartz glass. They emit mainly in the 185–254 nm range and therefore generate ozone when this light interacts with air, but it should not be forgotten that ozone is destroyed by 254 nm light.^12^ These lamps emit not only in the ultraviolet 100–400 nm range but also in the visible 380–700 nm range. This means that the emitted light interacts not only with air, water, and the photocatalyst but also with the MB dye.

**Figure 1:**
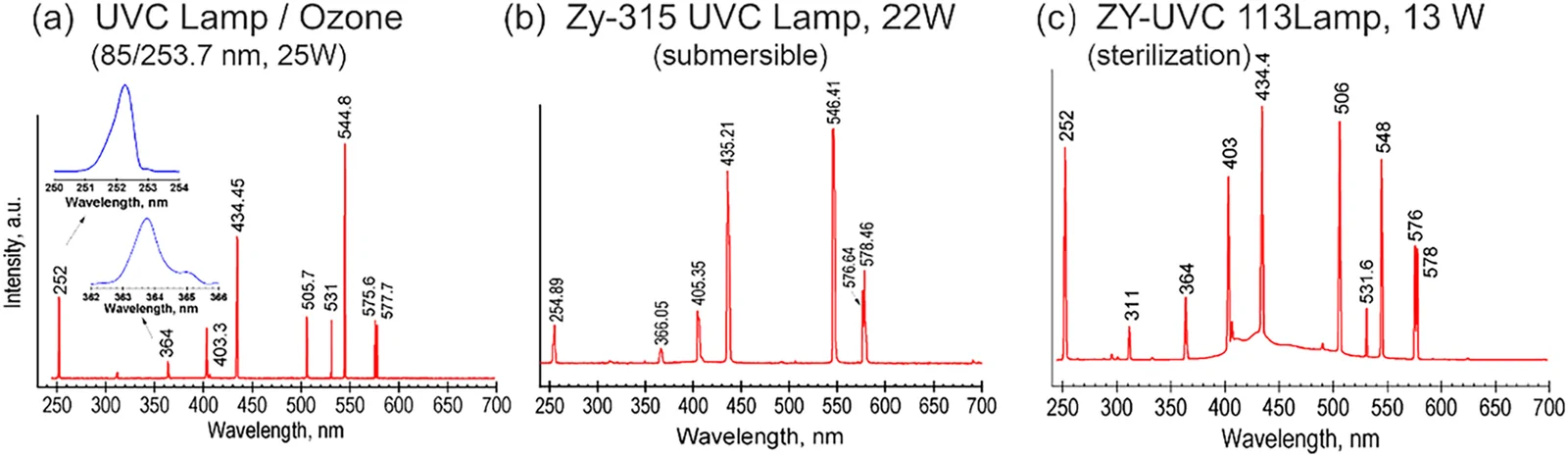
(a–c). Emission spectra of UVC lamps employed in this study. (a) Outdoor UVC ozone lamp (25 W), showing characteristic emission bands at 185 and 254 nm. (b) Submersible double UVC lamp (22 W). (c) Submersible UVC sterilization lamp (13 W).

### 2.2 Optical and Physicochemical Characterization

The quality of a liquid is determined by the concentrations of condensable and noncondensable gases, as well as the solutes it contains. Dissolved oxygen (DO) measurement data are used as an indicator of oxygen content in the liquid.^58–60^ Figure 2 summarizes the optical and structural characterization of MB and ZnO used in this study. The UV–vis absorption spectrum of MB dissolved in tap water (Figure 2a) exhibits the characteristic bands associated with monomeric and dimeric MB species.^16^ The optical response of cosmetic-grade ZnO powder dispersed in tap water is shown in Figure 2b. The absorption edge is typical of wide-band gap semiconductors, with a maximum peak observed at 376 nm. Additionally, the corresponding Tauc plot reveals the presence of multiple apparent band gap energies.^61^ The average *E*_g_ is found to be 3.13 ± 0.05 eV. Surface hydration, ZnO (HO-ZnO), and ion adsorption cause changes in surface tension and polarization. The particles of this material interact with light (*hv* > *E*_g_); oxygen and water molecules can interact with free electrons and holes, resulting in the generation of superoxide and OH radicals.^62–64^ The compositional analysis (EDX) of the ZnO powder, presented in Figure 2c, confirms the presence of Zn, O, and C at almost the same atomic percentage. This is due to the metal oxide forming a hexagonal wurtzite structure; the carbon fits into this structure as a substitutional defect without establishing a separate phase.^65^ The X-ray photoelectron spectroscopy (XPS) survey spectrum (Figure 2d) further supports the chemical purity of the sample and confirms the oxidation state of Zn. Earlier reports indicated that the O 1s state always contains three binding energy components: 530.15 eV and 531.25 are associated with O_2_^–^ ions, and the 532.40 eV peak is attributed to the chemisorbed oxygen.^66^ Also, the binding energy of 531.7 eV is attributed to chemisorbed or dissociated oxygen or hydroxyl (OH) species on the surface of the ZnO thin films.^67^ Morphological features of the ZnO powder are illustrated in the SEM image (Figure 2e), which reveals irregular, plate-like, and agglomerated structures; the particle size is nonuniform, ranging from approximately 150 nm to 2 μm, with an average of 0.7 ± 0.05 μm. Such morphologies enhance the effective surface area and may improve the interaction between ZnO particles, dissolved species, and reactive intermediates during cavitation and photocatalytic processes.^68,69^

**Figure 2:**
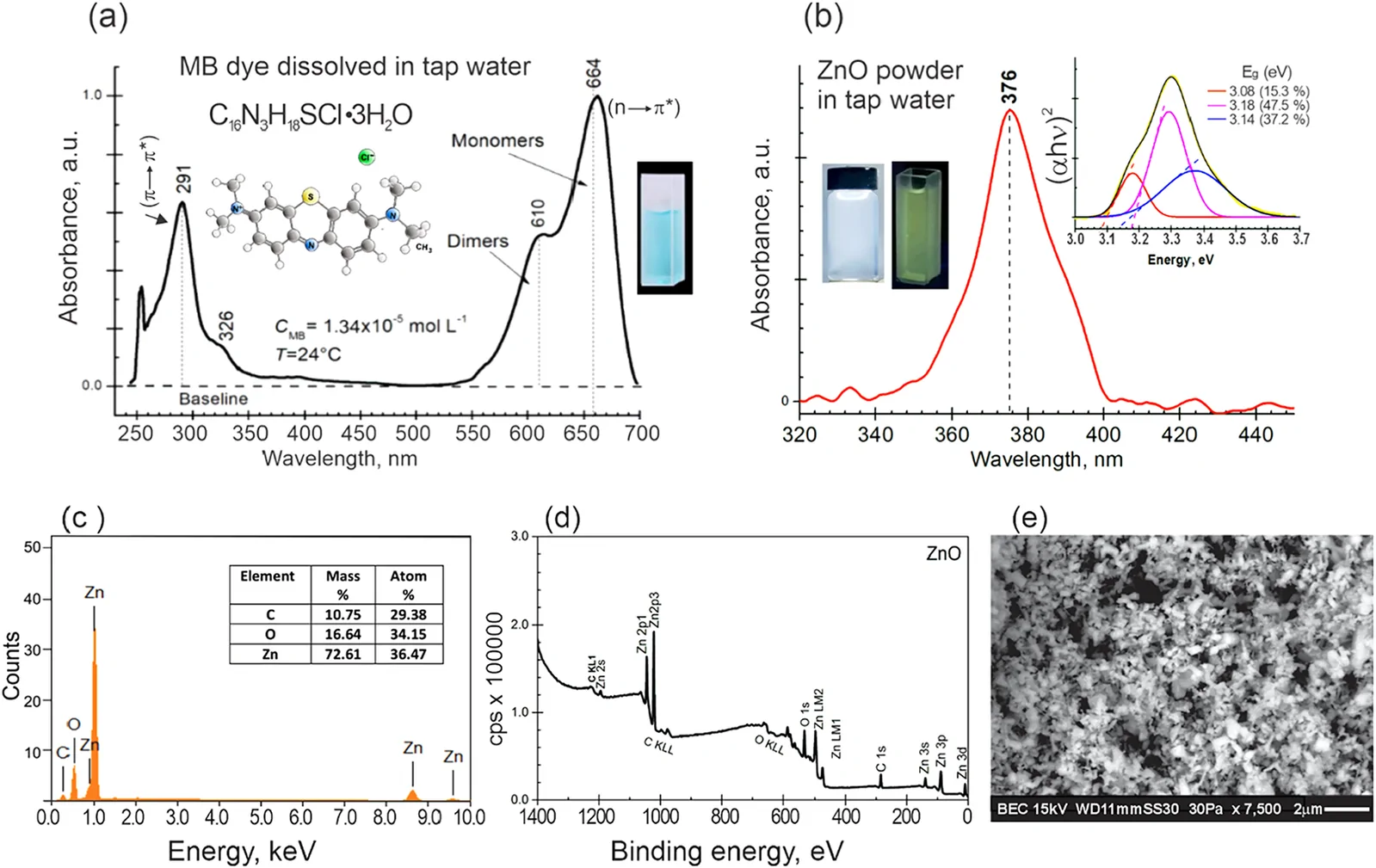
(a–e). Experimental characterization of methylene blue (MB) and ZnO. (a) UV–vis absorption spectrum of MB dissolved in tap water, showing the characteristic monomer and dimer bands. (b) UV–vis absorption spectrum of ZnO dispersed in tap water; inset: photographs of the colloidal suspension in quartz cuvettes under visible (left) and UVC (right) illumination. The upper inset shows the corresponding Tauc plot, indicating three optical band gap energies. (c) Energy-dispersive X-ray (EDX) spectrum of ZnO powder with the elemental composition summarized in the inset table. (d) X-ray photoelectron spectroscopy (XPS) survey spectrum of ZnO. (e) Scanning electron microscopy (SEM) image of the ZnO powder.

### 2.3 Electrochemical Response of the Aqueous Systems

Figure 3 shows the voltammograms acquired at a scan rate of 25 mVs^–1^ for tap water and deionized water, as well as for solutions with MB dye. Cyclic voltammetry measurements were performed at scan rates between 25 and 100 mVs^–1^, and the scan rate shown was selected based on signal stability and minimal noise. In Figure 3a, both cycles show no redox peaks in the scanned potential range, as expected due to the absence of electroactive species. Oxidation and reduction currents are larger for the tap water sample due to its higher conductivity and dissolved oxygen content. When MB dye is added to both solutions (Figure 3b), the curves present clear redox peaks. Table 2 summarizes the response of the electrochemical behavior of the MB dye in both matrix solutions: tap water and distilled water.

**Figure 3:**
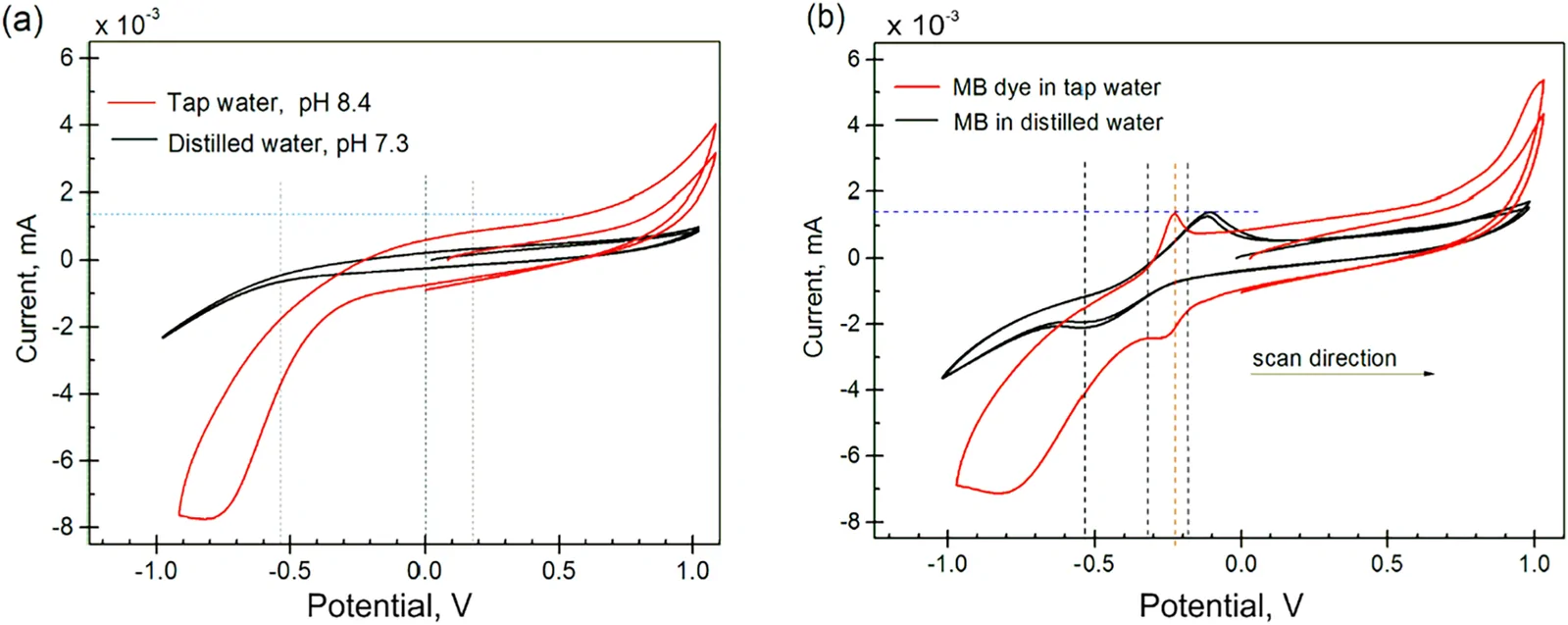
(a, b). Electrochemical characterization by cyclic voltammetry. (a) Voltammograms of tap water and deionized water. (b) Voltammograms of MB dissolved in tap and deionized water were recorded under identical experimental conditions. Potentials are reported for mercurous sulfate in saturated potassium sulfate (MSE, saturated K_2_SO_4_).

**Table 2:** Electrochemical Response from MB Dye as a Function of the Aqueous Matrix

Parameter	Observations
Peak current (*i*_p_)	Similar in both types of tested water, as expected. (Same concentration in both solutions)
Peak potential difference	Smaller (quasi-irreversible behavior) in tap water and larger in deionized water due to the resistivity of the solution (ohmic drop).No additional peaks due to MB dimerization were observed.
Peak shape	Wider in deionized water due to the ohmic drop.

### 2.4 Hydrodynamic System, Cavitation Regime, and Treatments

#### 2.4.1 Hydrodynamic Circuit

The experimental layout includes a recirculating hydraulic circuit with two main parts: a Venturi reactor with a relatively large throat and a controlled air inlet at atmospheric pressure regulated by a needle valve and a retention tank equipped with submersible UVC lamps. A feedback branch controls the volume and residence time of the aqueous solution inside the retention tank. This configuration ensures that the solution remains exposed to direct UVC irradiation for a longer period than the hydraulic cycle time, thereby improving the contact time between organic contaminants and oxidizing species. In addition, the system allows controlled gas injection and independent adjustment of cavitation and retention conditions, which is relevant for future scale-up and comparative process evaluation.

The hydraulic circuit depicted in Figure 4a includes a Venturi reactor, a centrifugal pump, two tanks, valves, measurement sensors, and a data acquisition system. In Figure 4b, the pump outlet volumetric flow-rate curve [Ls^–1^] is expressed as a function of the power consumption. A 2.5-in. schedule 80 PVC pipe directs the pump’s outlet flow. A Keyence-brand ultrasonic flowmeter, model FD-R50, is installed. Next, an elbow changes the flow direction; at this point, an air purge device is fixed. Subsequently, a section of schedule 40 PVC pipe is installed and connects to a 42 cm long quartz pipe. The circuit length is 7.94 m when valve 4 is closed. The 60 L retention tank has a water-cooling system, and the auxiliary tank is 300 L. The circuit has six globe valves to control the flow direction. Valve 4 controls the residence time of the aqueous volume inside the retention tank. The flow is sustained by a 5.5 hp centrifugal pump controlled by a variable frequency drive (Yaskawa J1000). A data acquisition system measures temperature and dissolved oxygen using a fiber optic sensor (Piccolo2, PyroScience) whose probe is installed inside the retention tank. The UVC lamps are positioned in three locations: two external lamps around the quartz pipe, which is connected to the Venturi inlet, and another located 5 cm above the water surface inside the recirculation tank. The other three submersible lamps are fixed in the middle of the retention tank, as shown in Figure 4a.

**Figure 4:**
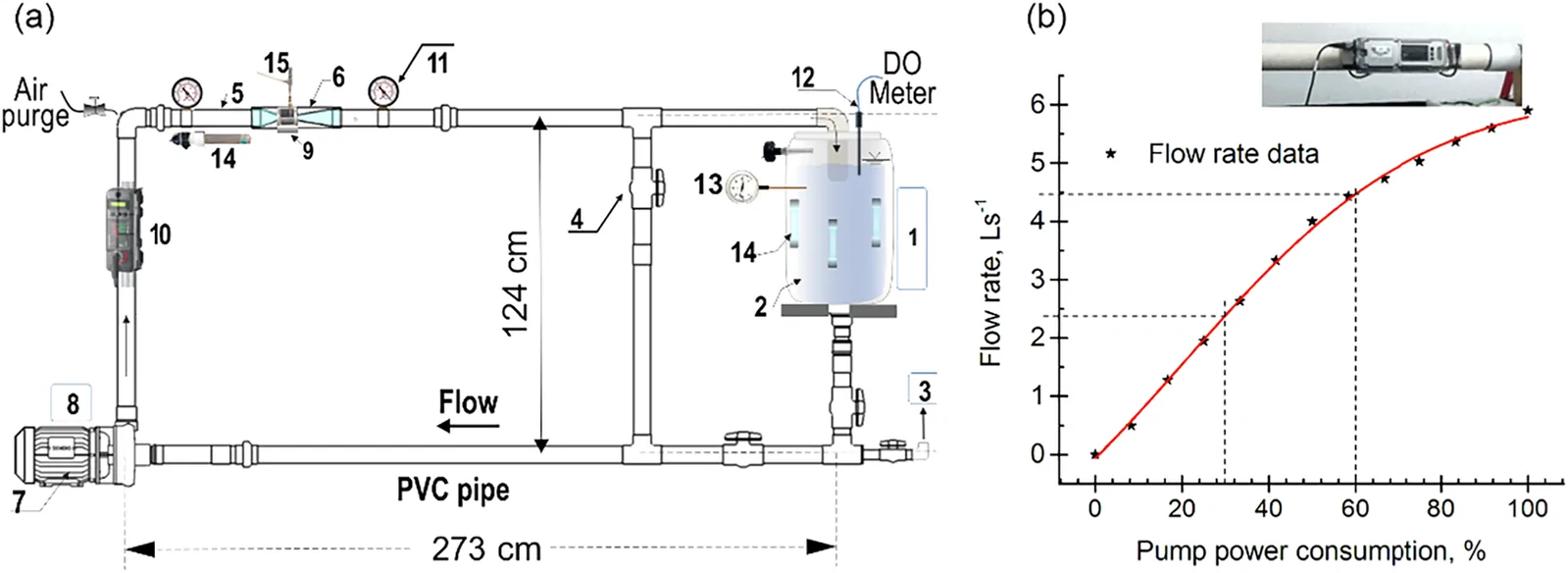
(a, b). Experimental setup and hydraulic performance. (a) Schematic diagram of the hydraulic circuit used in the experiments. The labeled components correspond to (1) water cooling system, (2) retention tank, (3) storage tank, (4) control valve, (5) quartz pipe, (6) Venturi reactor, (7) centrifugal pump, (8) variable frequency drive, (9) piezoelectric transducer, (10) ultrasonic flowmeter, (11) pressure gauges, (12) fiber-optic (DO) sensor, (13) digital thermometer, (14) UVC lamps, and (15) needle valve for air inlet to the throat. (b) Hydraulic circuit flow rate as a function of pump power consumption without Venturi. Local tap water was used as the working fluid.

#### 2.4.2 Venturi Reactor

Figure 5a shows the schematic of the Venturi tube with its geometric and dynamic characteristics. The inlet and outlet diameters are 64 mm each. The throat section has an L/D ratio equal to 4; its long path guarantees heat transfer and visualization of the vaporization process. Figure 5b shows the pressure profile.^55–57^ Figure 5c shows the cavitation number (σ) evolution in the Venturi tube as a function of the power consumed by the pump. This explicit hydrodynamic characterization of the Venturi stage, including pressure profile and cavitation- number evolution, provides a better-defined basis for comparing the reactor performance under different treatment conditions and supports the intended application-oriented design of the system.

**Figure 5:**
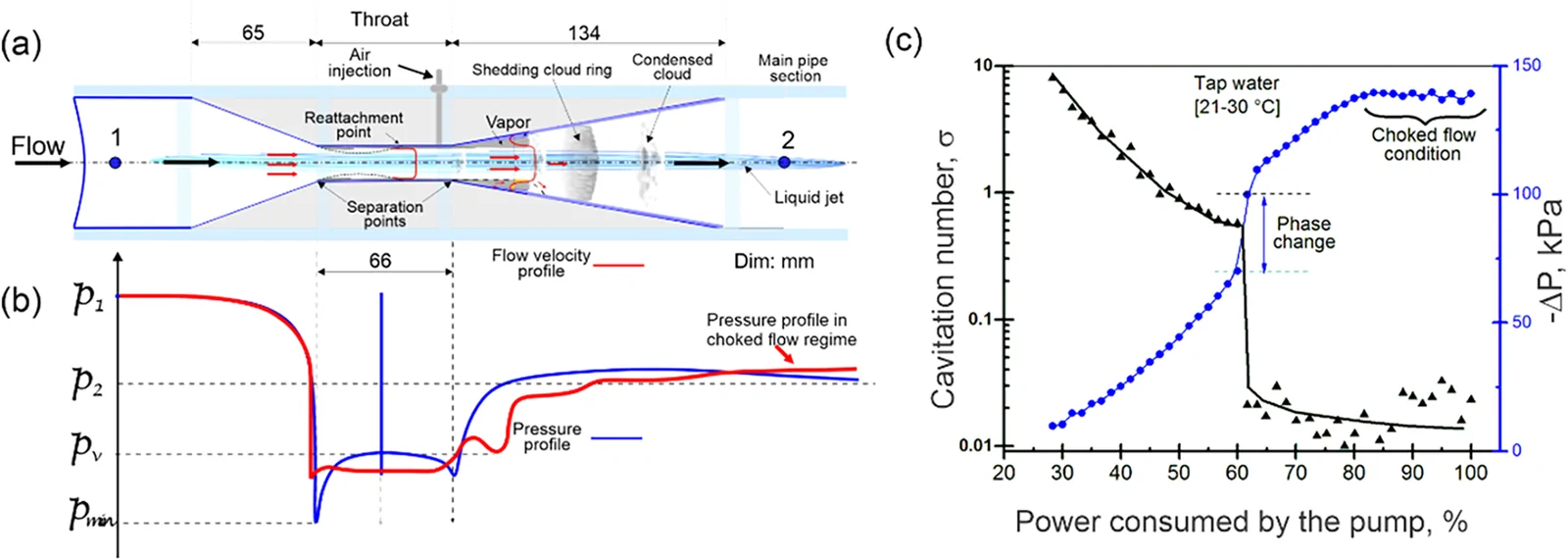
(a–c). Venturi reactor and cavitation behavior. (a) Geometrical configuration of the Venturi reactor, relevant dimensions, and identification of different cavitation stages as a function of Venturi length. (b) Pressure profile along the Venturi reactor. The pressure at the inlet of the convergent section is denoted as “*p*_1_”, the outlet pressure as “*p*_2_”, the vapor pressure as “*p*_v_”, and the minimum pressure as “*p*_min_”. (c) Cavitation number (σ) and pressure drop in the Venturi tube (−Δ*P*) as a function of pump power consumption, showing the onset of choked-flow conditions at pump powers above approximately 80%.

#### 2.4.3 Treatments for the Degradation of MB Dye in Tap Water Solution

The aqueous mixture was recirculated at low speed in the hydraulic circuit by setting the pump to 10% power for 15 min. Each trial uses a maximum of 70 L of aqueous mixture to fill the hydraulic circuit; the mixture contains 150 mg of MB powder, and, for the photocatalysis configuration, 3.0 g of zinc oxide powder is added. To produce laminar flow, the pump power is maintained at 30% of its capacity. To generate vaporous cavitation, the pump operates at 80% (choked flow with the air inlet valve closed). This process generates cyclic cavitation with σ ≈ 0.3. To generate σ ≈ 0.7 in the Venturi throat, the pump power is set at 60%, and the air inlet valve is open.

The hydrodynamic circuit, with and without the reactor, was used to test and analyze the initial operating parameters, including ZnO/UVC without cavitation and dissolved-oxygen-controlled experiments, as well as different concentrations of MB and ZnO powder. From these preliminary, cavitation-only tests showed that the dissolved oxygen decreased in the retention tank and that MB degradation was negligible after 1 h. Therefore, an air inlet at the Venturi throat was implemented to control dissolved oxygen in the aqueous mixture. When the dye concentration exceeds 20 mg/L, cavitation literature commonly recommends oxygen injection or addition of superoxidants such as peroxides (H_2_O_2_), ozone, halogens, etc.^29,30,37,41^

Table 3 summarizes the operating parameters for four treatments. The aim is to assess how coupling hydrodynamic cavitation (HC) with photolysis or photocatalysis improves the degradation efficiency of methylene blue dissolved in tap water. For clarity, the experimental design includes two baseline control conditions. Hydrodynamic vaporous cavitation was used as the cavitation-only control, while dynamic flow-UVC photolysis was used as the UVC-only control under noncavitating flow conditions. These reference configurations provide the basis for evaluating the enhancement observed when hydrodynamic cavitation was coupled with UVC photolysis or UVC-ZnO photocatalysis.

**Table 3:** Treatments for the Degradation of MB Dye in Tap Water Solution^a^

Configurations	Pump Power [%]	Flow Type	ZnO [g]	σ
Hydrodynamic vaporous cavitation	80	Turbulent	0	0.3
Dynamic flow-UVC photolysis	30	Laminar	0	6
⧫HC-UVC photolysis	60	Turbulent	0	0.7
⧫HC-UVC-ZnO photocatalysis	60	Turbulent	3	0.7

a ⧫ Aerated; HC = hydrodynamic cavitation; σ = cavitation number.

Each condition in Table 3 corresponds to a single long-duration run (up to ∼3000 min); therefore, replicate-based error bars are not included. The fitting quality is documented through the *R* ^2^ values reported for the pseudo-first-order analysis. Accordingly, the reported *k* values are interpreted as apparent, condition-specific parameters used for comparative purposes among the four configurations.

## 3 Experimental—Treatments Effects

### 3.1 Hydrodynamic Vaporous Cavitation

The pump operates at 80% power with no air injected into the Venturi throat; the retention tank remains open to the atmosphere. The circulating solution forms vapor clouds that cyclically detach, advance, and collapse in higher-pressure zones.^54,55,57^ As cavitation proceeds in the choked flow region (Figure 5c), dissolved oxygen in the turbulent solution decreases (retention tank), while the vapor content rises (Figure 6a); the color changes, and the absorption spectrum is shown in Figure 6b. After 3000 min (50 h) of processing, degradation efficiency reaches 68% (Figure 6c). Figure 6d shows numerical analysis images based on experimental data: vaporous cavitation is achieved at the Venturi diffuser and expands into the pipe. Within the Venturi, a steady dynamic cycle is established: a nonvaporizing liquid layer clings to the throat edge while a central ring-shaped cloud forms around the main flux, densifies, detaches, and collapses in the downstream high-pressure region. These observations also illustrate the usefulness of the present circuit configuration for separating cavitation effects from retention-time and irradiation effects, which is relevant for the comparative evaluation of hybrid HC-assisted treatments.

**Figure 6:**
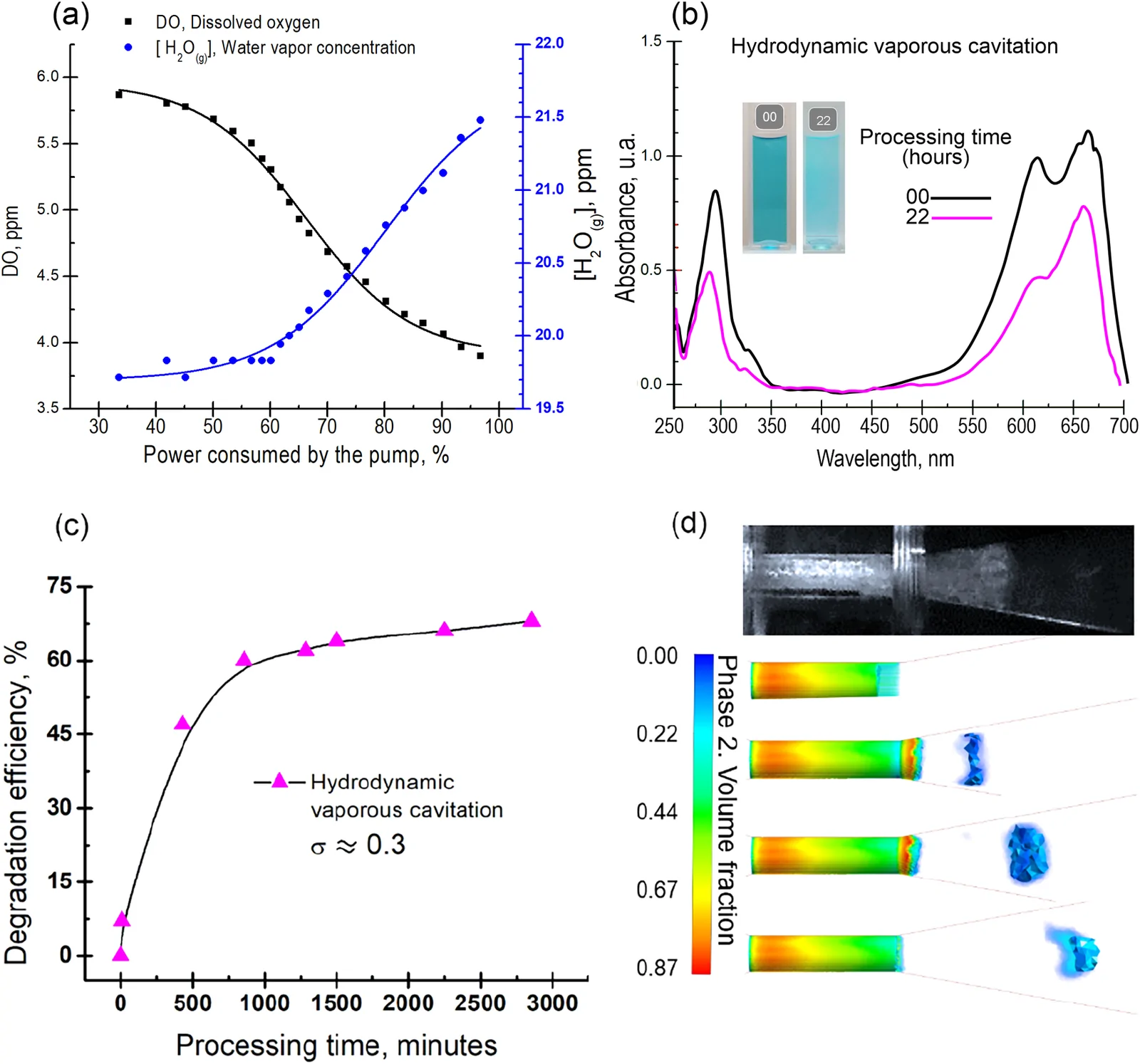
(a–d). Hydrodynamic vaporous cavitation. (a) Dissolved oxygen concentration drops and increases in water vapor concentration as a function of pump power consumption. (b) UV–vis absorption spectra recorded at the beginning of the process and after 1320 min (22 h) of cavitation treatment. (c) Degradation efficiency as a function of processing time conducted at σ ≈ 0.3. (d) Numerical analysis based on experimental data, illustrating the cyclic formation and transport of vaporous cavities reaching the Venturi diffuser. The images in panel d were provided courtesy of Javier A. Rosas. Copyright 2025.

### 3.2 Dynamic Flow-UVC Photolysis

Experiments were conducted under a noncavitating regime: the solution circulated without bubble formation in the Venturi throat, operating at 30% and without air injection. Under this regime, the dissolved oxygen concentration decreased over time (Figure 6a). Water photolysis generates H^+^, ^•^OH, O_2_, and H_2_ via direct photon decomposition; the redox mechanisms are discussed in many references.^10,13–17^ Figure 7(a–c) summarizes the observations: (a) color evolution at selected times; (b) UV–vis absorption spectra recorded hourly; (c) degradation efficiency profile. Processing lasted 12 h and was performed intermittently (one hour of operation followed by cooling) to maintain the recirculation tank at 18–25 °C. After eight hours of processing, the dye concentration decreases by 90%, and subsequently, the color remains practically constant.

**Figure 7:**
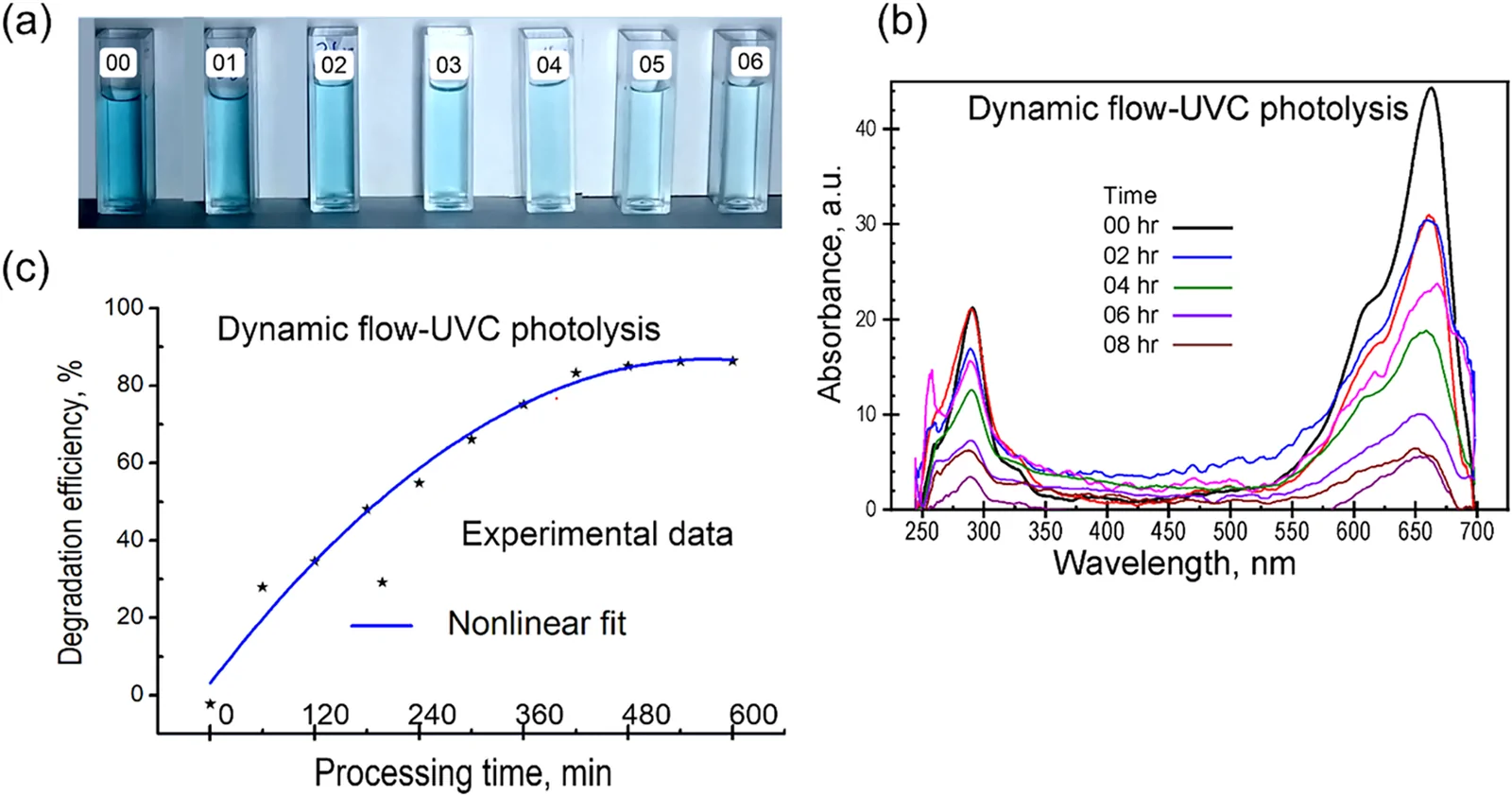
(a–c). Dynamic flow-UVC photolysis. (a) Photograph showing the color evolution of the solution at different processing times. (b) UV–vis absorption spectra recorded at hourly intervals during the dynamic flow photolysis process. (c) Degradation efficiency as a function of processing time.

### 3.3 HC-UVC Photolysis

The MB-water solution flow is maintained in cavitation mode in the Venturi reactor, operating at 60%, σ ≈ 0.7, Re ≈ 55,000, and air injection at the throat. Under this regime, the dissolved oxygen in the volumetric mass flow remains constant. In cavitation, the bubble cluster undergoes nonlinear oscillations between the throat and the diffuser, as described in Figure 6d. Under photolysis, water yields reactive intermediates—primarily H^•^ and ^•^OH—from which H_2_ and O_2_ arise via subsequent recombination and redox pathways. The reactions and their mechanisms have been analyzed and discussed at length.^10,13–17,71^ This process is applied for 8 h using the same methodology as the previous processes described above. Figure 8 summarizes the observations: (a) color evolution at selected times; (b) UV–vis absorption spectra recorded hourly; (c) degradation efficiency profile. After six hours of processing, the color intensity decreases by 98%, and subsequently, the color remains practically constant.

**Figure 8:**
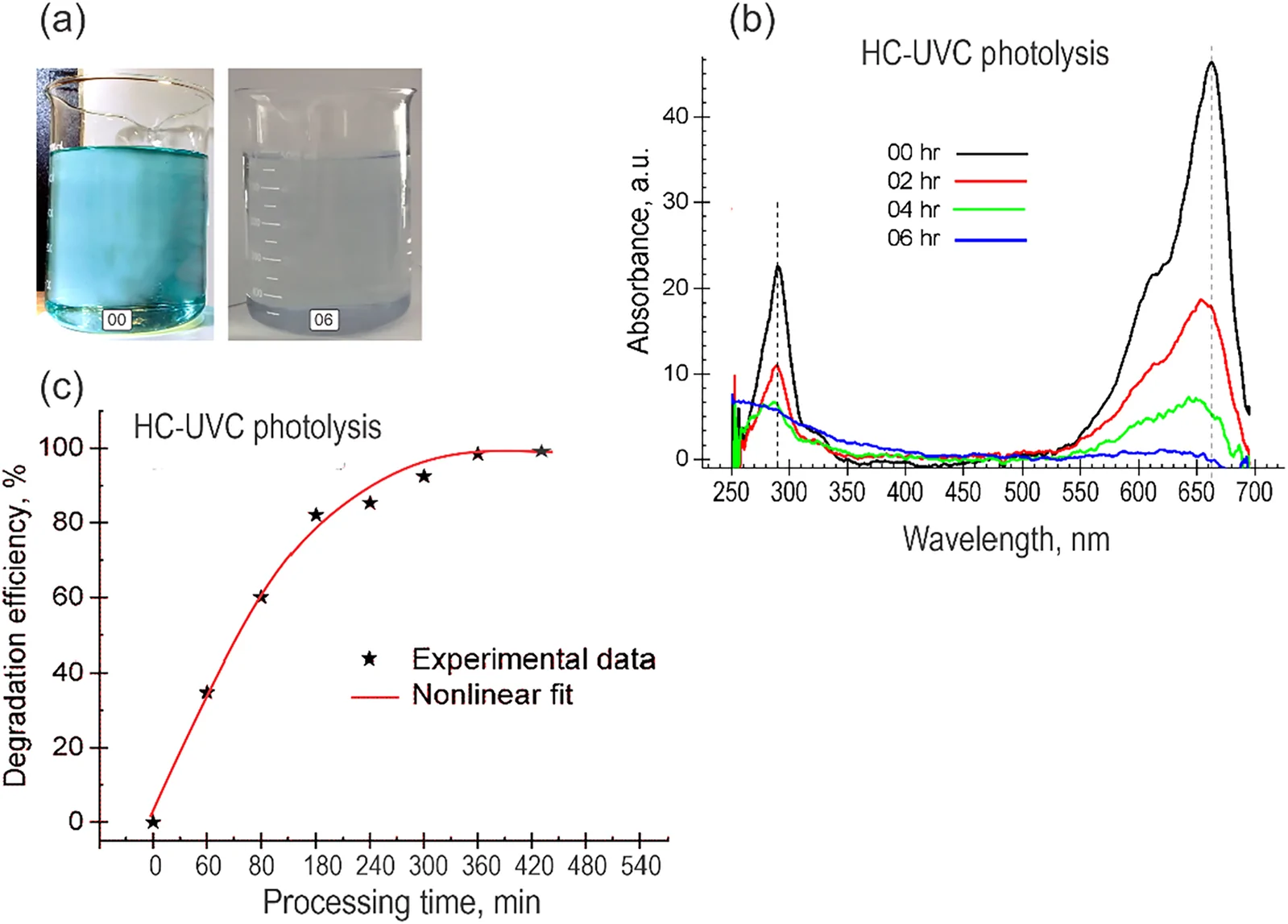
(a–c). HC-UVC photolysis. (a) Photographs showing the color evolution for 0 and 6 h of treatment. (b) UV–vis absorption spectra collected at different times during the hydrodynamic cavitation-photolysis process. (c) Degradation efficiency as a function of processing time; symbols correspond to experimental data, and the solid line represents the nonlinear fit.

### 3.4 HC-UVC-ZnO Photocatalysis

The MB-tap water solution containing ZnO powder is maintained in cavitation mode with a pumping power of 60%, producing a σ ≈ 0.7 and a Reynolds number of ≈ 55,000. In this regime, a liquid film adheres to the throat walls, while a surrounding layer of water vapor bubbles develops with a core flow that continues downstream. The resulting bubble cluster undergoes nonlinear oscillations between the throat and the diffuser, as illustrated in Figure 6d. Under this condition, the dissolved oxygen (DO) concentration is almost constant throughout the mass flow rate. The degradation process using this method lasted two hours and, similar to the previous approach, was conducted in stages. Figure 9a shows the color change during the process. In Figure 9b, the absorption spectra suggest complete disappearance of the dye; however, this apparent total removal reflects the detection limit of the spectrometer rather than total elimination. Although HC-UVC-ZnO photocatalysis provided the fastest discoloration of MB in tap water, the treated solution may still contain residual ZnO-related material, indicating a potential secondary contamination concern.

**Figure 9:**
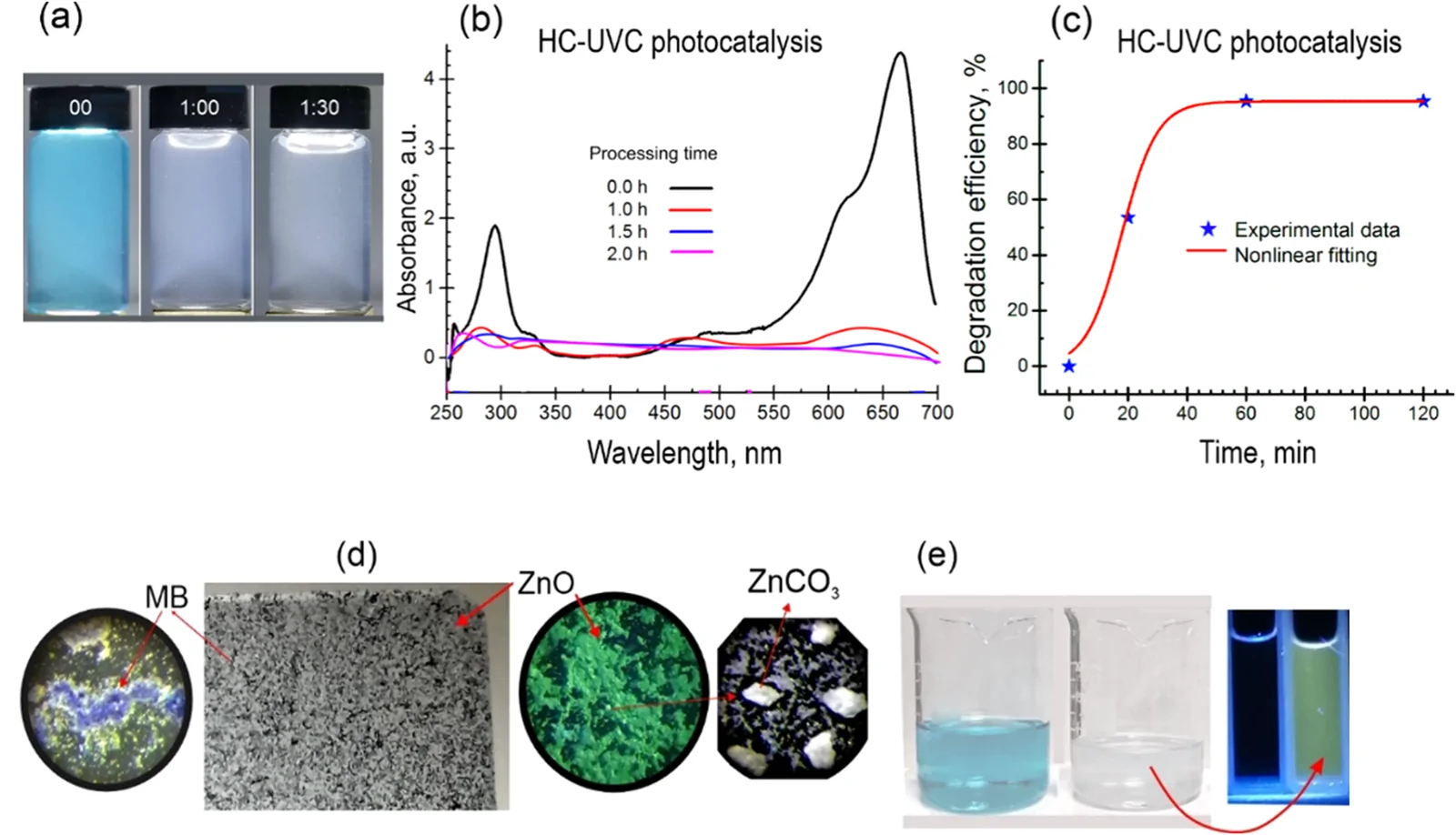
(a–e). HC-UVC photocatalysis. (a) Photographs illustrating the progressive discoloration of the MB-ZnO suspension in tap water during treatment. (b) UV–vis absorption spectra collected from a midvolume aliquot at different processing times, showing a decrease in the dye concentration. (c) Degradation efficiency as a function of processing time. (d) Optical-microscopy images of ZnO-MB residues obtained after evaporation of the liquid mixture on a glass slide at ambient temperature: right, visible-light image showing MB aggregates with dendritic morphology and ZnO structures; left, image under 365 nm LED excitation highlighting ZnCO_3_ agglomerates. (e) Images of the treated solution after 3 days of rest, showing green-yellow emission under 365 nm excitation, compared with tap water.

Finally, Figure 9c presents the degradation process, which occurs at a faster rate compared with the previous processes, achieving approximately 98% efficiency. Subsequently, a 100 mL aliquot was cast onto glass slides and allowed to evaporate at room temperature, producing a porous deposit, with almost constant thickness, of agglomerates and large crystals (Figure 9d).

The ZnO powder appears to absorb only a limited amount of MB, forming plaques and dendritic clusters. Large granules were absent in samples prepared before applying this process; see the left image in Figure 9d. Under 365 nm illumination, these granules emit a bluish light, clearly distinct from the greenish-yellow emission of the surrounding powder; effervescence upon application of diluted HCl supports the presence of zinc carbonate phases (e.g., hydrozincite, Zn_5_(CO_3_)_2_(OH)_6_). Figure 9e shows the photoluminescence of the processed aqueous mixture: the green-yellow emission is visible to the naked eye and is compared with tap water alone under 365 nm LED excitation.

## 4 Results

### 4.1 Comparison of MB Degradation in Tap Water in the Presence of HC

Figure 10. (a) Degradation efficiency of MB as a function of processing time for the four configurations. (b) Representation of normalized concentration decay profiles vs the extended processing times required per condition, with hydrodynamic vaporous cavitation alone reaching nearly 3000 min. Given the long processing times required per condition, replication data sets were not collected, and thus, error bars are not shown. Accordingly, the pseudo-first-order fits are reported with *R* ^2^ values to document fitting quality and to support comparison of the apparent rate constant across configurations; also, all operation parameters were kept strictly constant to ensure consistency across experiments. The degradation efficiency profiles (Figure 10a) show clear differences in the rate and extent of degradation, reflecting the dominant physicochemical mechanisms activated in each process. Hydrodynamic vaporous cavitation exhibits the slowest degradation, even with a cavitation number close to 0.3. In contrast, coupling dynamic (nonturbulent) flow with UVC photolysis improved the degradation efficiency, consistent with the contribution of direct photochemical pathways. An increase in degradation rate is observed when hydrodynamic cavitation (HC) is coupled to UVC photolysis (σ ≈ 0.7 with aeration). This highlights the synergistic role of the oxidizing species generated by cavitation and the reactions that occur upon exposure to UVC light. The highest degradation rate is achieved when HC is coupled with UVC-ZnO photocatalysis, as evidenced by the marked increase in degradation efficiency in a short processing time. To quantitatively compare the degradation rates across configurations, the concentration decay was analyzed using a pseudo-first-order kinetic model, where k is an apparent rate constant. This approach is used here as a practical and widely adopted comparative metric to rank process intensification under identical initial concentration and operating conditions, rather than to claim a fully mechanistic kinetic description. Because of the extended processing times, the experimental degradation curves deviate from ideal first-order behavior and tend toward saturation under the applied conditions. We attribute this plateau to a combination of factors, such as (i) the progressive decrease in residual dye concentration, which reduces the probability of reaction with oxidizing radicals; (ii) the formation of intermediate products that compete for reactive species; (iii) radical–radical recombination that lowers the effective ^•^OH availability; and (iv) mass-transfer limitations that restrict dye transport to highly active zones within the system (including possible adsorption or retention on reactor walls and connecting lines). Therefore, the pseudo-first-order constants reported in Figure 10b should be interpreted as apparent, condition-specific parameters useful for comparative purposes, particularly in the initial-to-intermediate degradation regime before the onset of saturation.

**Figure 10:**
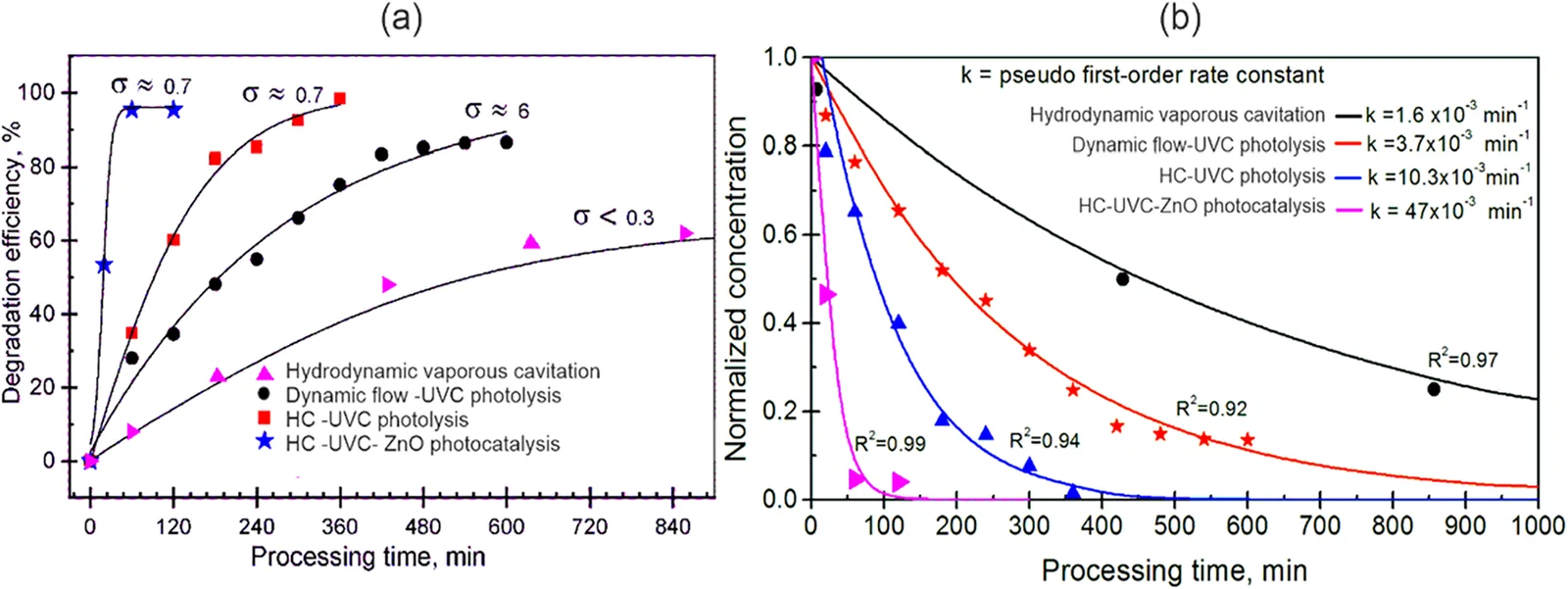
(a, b). (a) Degradation efficiency of MB as a function of processing time for the four investigated configurations. (b) Pseudo-first-order representation of the normalized concentration decay as a function of time. The apparent rate constant, *k*, was estimated from the linear fits, and the corresponding coefficients of determination (*R* ^2^) are indicated for each case.

### 4.2 Electrochemical and Luminescence Response of the Solution after HC-UVC-ZnO

Figure 11 addresses the detection of residual ZnO nanoparticles remaining in the treated aqueous solution after the application of HC-UVC photolysis and HC-UVC-ZnO photocatalysis. The cyclic voltammograms recorded after both processes (Figure 11a) show nearly identical electrochemical responses, with no discernible features between them. These results indicate that, within the sensitivity limits of the electrochemical setup employed, residual photocatalyst particles cannot be detected in the processed water. Similarly, spectroscopic techniques applied directly to the aqueous phase did not provide clear evidence of remaining ZnO nanoparticles. In contrast, photoluminescence performed on aqueous samples before and after HC-UVC-ZnO photocatalysis (Figure 11b) reveals emission features consistent with ZnO-related species, providing qualitative evidence of residual photocatalyst-related material in suspension that was not resolved by voltametric or conventional optical absorption measurements. However, this observation should be regarded as qualitative, since no dedicated elemental quantification or fractionation analysis was performed in the present study. These results indicate that HC-UVC-ZnO photocatalysis degrades methylene blue rapidly and effectively but may also leave residual ZnO-related material in the treated water. Therefore, while this hybrid process improves degradation performance, it also raises an important environmental consideration regarding possible secondary contamination.

**Figure 11:**
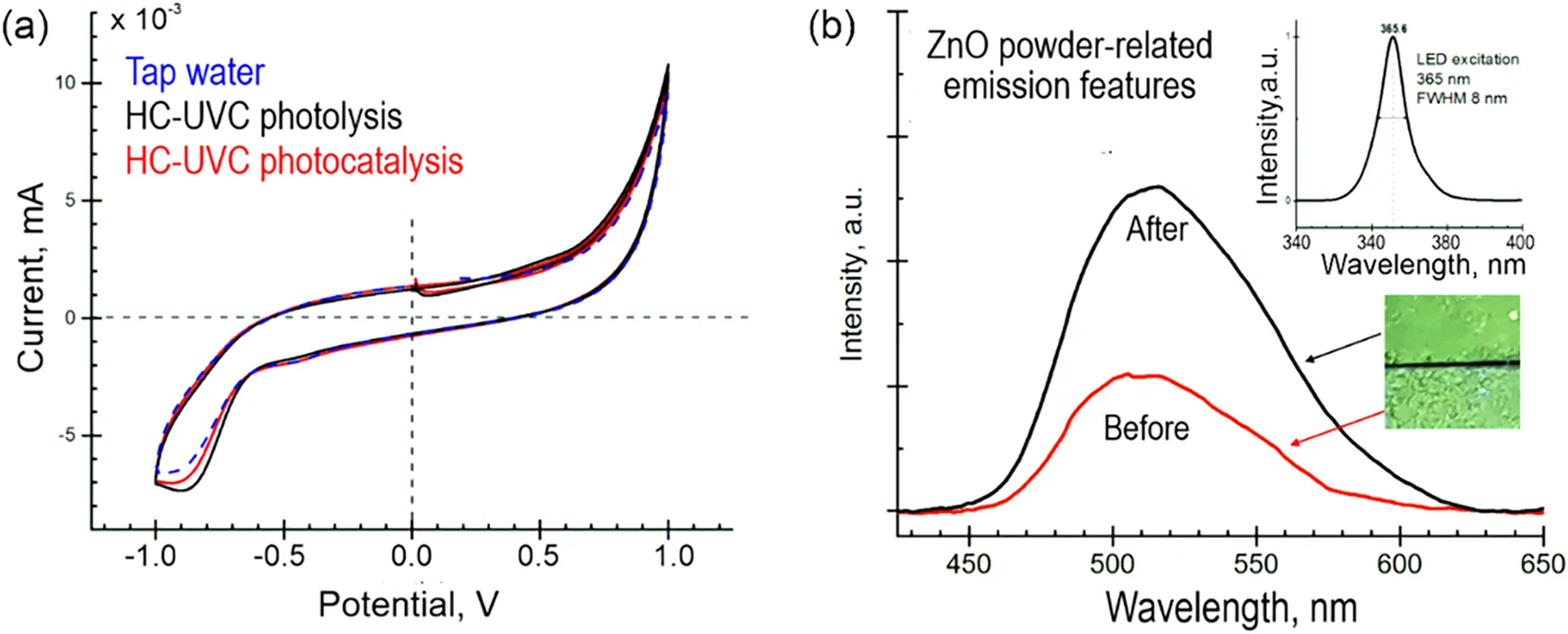
(a, b). Detection limits of residual ZnO nanoparticles after HC-UVC treatment. (a) Comparison of cyclic voltammograms recorded from aqueous solution after hydrodynamic coupling with UVC photolysis and UVC photocatalysis. (b) Photoluminescence spectra of porous deposited precipitates recovered from aqueous samples before and after treatment, showing ZnO-related emission features. The observed emission is presented as qualitative evidence consistent with residual ZnO-related material after treatment.

### 4.3 Water Quality Parameters

As discussed in the water matrix section, the tap water was assessed through bulk physicochemical parameters rather than full compositional speciation. Table 4 summarizes the water quality parameters after each degradation process, including temperature, total dissolved solids (TDS), electric conductivity (EC), pH, salt percentage, degradation efficiency, processing time, the corresponding electrical energy per order (*E*_EO_) and synergetic index (ξ). The *E*_EO_ values reported here include pumping and irradiation energy during the treatment stage but do not include the additional energy and operational cost required for downstream ZnO separation/recovery.

**Table 4:** Summary of Water Quality Measures, Degradation Efficiency, and Energy Metrics Subsequent to the Application of Each Treatment^a^

Aqueous Media and Type of Treatment	σ	TDS [ppm]	EC [μS cm^–1^]	pH	Salt [%]	Time [h]	Deg [%]	*k* [min^–1^]	E_EO_ [kWhm^–3^]	Syn index (ξ)
Tap water		337	680	8.6	0.03					
Distilled water		26	5.3	7.8	0					
Deionized water		1.3	0.07	6.98	0					
Hydrodynamic vaporous cavitation	0.3	350	685	8.6	0.03	50	68	0.0016	679	
Dynamic flow-UVC photolysis	6	362	727	8.7	0.03	8	90	0.0037	155	
⧫HC-UVC photolysis	0.7	361	728	8.8	0.03	6	98	0.0103	221	1.94
⧫HC-UVC-ZnO photocatalysis	0.7	338	682	8.5	0.03	<1	98	0.0470	38^b^	1.88

a The temperature of the dye solution was maintained between 20 and 27 °C.

b The reported energy consumption does not include downstream photocatalyst recovery. ⧫ Aerated conditions.

At first glance, the HC-UVC-ZnO photocatalysis configuration appears to provide the best overall performance. However, this is not the case, since practical implementation of this process would require an additional post-treatment step to remove residual ZnO-related material remaining in suspension. As discussed above, this residual material was only qualitatively detected by photoluminescence and was not resolved by TDS measurements, voltametric response, or direct absorption analysis. In practice, ZnO powder recovery would likely require membrane-based separation and/or adsorption-based recovery, which would increase both system complexity and operational cost. Therefore, when sludge generation and catalyst recovery are undesirable, the HC-UVC photolysis configuration with air injection may represent the more practical option, as it provides high degradation efficiency without requiring downstream recovery of a semiconductor photocatalyst.

### 4.4 Discussion

Textile effluents containing synthetic dyes represent a persistent environmental challenge due to their chemical stability, limited biodegradability, and potential formation of toxic intermediates during incomplete degradation. In particular, the degradation of methylene blue (MB) may generate aromatic amines that can exhibit higher toxicity than the parent compound, emphasizing the need for treatment strategies that promote rapid and effective oxidation while minimizing byproduct formation.^64^ It has been reported that the MB degradation pathway is strongly influenced by the physicochemical properties of water matrix, which in the present case may act as a weak buffer. Furthermore, MB degradation also depends on the initial concentration of the dye and the availability of dissolved oxygen. Electrochemical analyses highlighted a pronounced matrix effect, while dissolved oxygen is identified as a key parameter controlling the efficiency of radical-driven oxidation processes. These factors represent fundamental design constraints when evaluating individual or coupled treatment technologies.

Among the dynamic treatment configurations, hydrodynamic vaporous cavitation alone exhibited limited degradation efficiency and slow kinetics, primarily due to confinement of the dye in the liquid phase and a progressive depletion of dissolved oxygen. The main mechanism of this process is explained in previous studies,^26–28,30,37,40,43^ which discuss the formation of radicals along with high turbulence and shear mixing. Additional oxidants are added to achieve high dye degradation.^36,39,42,45^ Previous studies have demonstrated that hydrodynamic cavitation and photocatalytic or hybrid advanced oxidation systems can generate highly oxidative radical species, particularly ^•^OH; however, in the absence of dedicated scavenging or ESR/EPR experiments, the specific dominant reactive species in the present system cannot be conclusively assigned.^72–74^

Other devices that generate hydrodynamic cavitation include rotating discs (RGHC).^50,70^ Additionally, the pressure in the hydraulic circuit can be increased.

Coupling cavitation with photolysis or photocatalysis significantly enhanced degradation rates, reflecting synergistic interactions between hydrodynamic, photochemical, and catalytic mechanisms. In particular, hydrodynamic cavitation coupled with photocatalysis achieved the highest degradation efficiency, fastest kinetics, and lowest electrical energy per order, as summarized in Table 4.

Despite its superior performance, the photocatalysis-assisted configuration introduces a practical limitation associated with residual ZnO nanoparticles remaining in the treated water. While electrochemical and absorption techniques failed to detect these residues, photoluminescence analysis confirmed their presence. This finding underscores a potential trade-off between process efficiency and system simplicity, suggesting that, in some applications, hydrodynamic cavitation coupled with photolysis—despite requiring longer processing times—may offer a more robust and economically viable solution by avoiding the need for downstream photocatalyst recovery.

## 5 Conclusions

This study demonstrates that hydrodynamic cavitation provides an effective platform for the degradation of methylene blue in aqueous systems and that its performance can be significantly enhanced through coupling with photolysis and photocatalysis. Comparative analysis of degradation efficiency, kinetic constants, and electrical energy per order revealed strong synergistic effects when hydrodynamic cavitation is combined with photonic and catalytic processes. Among the evaluated configurations, HC-UVC-ZnO photocatalysis exhibited the fastest degradation kinetics and the lowest energy consumption, highlighting its potential as a highly efficient advanced oxidation strategy. However, photoluminescence analysis revealed the presence of residual ZnO nanoparticles at concentrations below the detection limits of commonly used analytical techniques, indicating a practical limitation related to downstream catalyst recovery. In contrast, HC-UVC photolysis, while requiring longer processing times, avoids the introduction of solid photocatalysts and offers a simpler and potentially more robust system configuration. When long-term operation, maintenance requirements, and overall process simplicity are considered, this approach may represent a more economically viable option for certain water treatment applications. Overall, these findings emphasize that optimal process selection should not rely solely on degradation efficiency but should consider an integrated assessment of kinetics, energy consumption, system simplicity, and post-treatment requirements.

## References

[1] Pereira, L. ; Alves, M. Dyes–Environmental Impact and Remediation. In Environmental Protection Strategies for Sustainable Development; Springer, 2011; Chapter 15; pp 111–162 10.1007/978-94-007-1591-2_4.

[2] Katheresan V., Kansedo J., Lau S. Y. (2018). Efficiency of Various Recent Wastewater
Dye Removal Methods: A Review. J. Environ. Chem.
Eng..

[3] Dutta P., Rabbi M. R., Sufian M. A., Mahjebin S. (2022). Effects of Textile
Dyeing Effluent on the Environment and Its Treatment: A Review. Eng. Appl. Sci. Lett..

[4] Slama H. B., Bouket A. Ch., Pourhassan Z., Alenezi F. N., Silini A. (2021). Diversity of Synthetic
Dyes from Textile Industries, Discharge Impacts
and Treatment Methods. Appl. Sci..

[5] Halepoto H., Gong T., Memon H. (2022). Current Status and Research Trends
of Textile Wastewater Treatments—A Bibliometric-Based Study. Front. Environ. Sci..

[6] Forgacs E., Cserháti T., Oros G. (2004). Removal of Synthetic
Dyes from Wastewaters:
A Review. Environ. Int..

[7] Dutta S., Gupta B., Srivastava S. K., Gupta A. K. (2021). Recent Advances
on the Removal of Dyes from Wastewater Using Various Adsorbents: A
Critical Review. Mater. Adv..

[8] De
Gisi S., Lofrano G., Grassi M., Notarnicola M. (2016). Characteristics
and Adsorption Capacities of Low-Cost Sorbents for Wastewater Treatment:
A Review. Sustainable Mater. Technol..

[9] Ma D., Yi H., Lai C., Liu X., Huo X., An Z., Li L., Fu Y., Li B., Zhang M., Qin L., Liu S., Yang L. (2021). Critical Review
of Advanced Oxidation Processes in
Organic Wastewater Treatment. Chemosphere.

[10] Kumari P., Kumar A. (2023). Advanced Oxidation
Process: A Remediation Technique for Organic and
Non-Biodegradable Pollutant. Results Surf. Interfaces.

[11] Hübner U., Spahr S., Lutze H., Wieland A., Rüting S., Gernjak W., Wenk J. (2024). Advanced Oxidation
Processes for
Water and Wastewater Treatment -Guidance for Systematic Future Research. Heliyon.

[12] Pandis P. K., Kalogirou C., Kanellou E., Vaitsis C., Savvidou M. G., Sourkouni G., Zorpas A. A., Argirusis C. (2022). Key Points
of Advanced Oxidation Processes (AOPs) for Wastewater, Organic Pollutants
and Pharmaceutical Waste Treatment: A Mini Review. ChemEngineering.

[13] Buxton G. V., Greenstock C. L., Helman W. P., Ross A. B. (1988). Critical Review
of Rate Constants for Reactions of Hydrated Electrons, Hydrogen Atoms,
and Hydroxyl Radicals (^•^OH/^•^O−)
in Aqueous Solution. J. Phys. Chem. Ref. Data.

[14] Ribeiro A.
R. L., Moreira N. F. F., Puma G. L., Silva A. M. T. (2019). Impact
of Water Matrix on the Removal of Micropollutants by Advanced Oxidation
Technologies. Chem. Eng. J..

[15] Miklos D. B., Remy C., Jekel M., Linden K. G., Drewes J. E., Hübner U. (2018). Evaluation of Advanced Oxidation
Processes for Water
and Wastewater Treatment—A Critical Review. Water Res..

[16] Fernández-Pérez A., Marbán G. (2020). Visible Light Spectroscopy Analysis of Methylene Blue
in Water: What Comes after Dimer?. ACS Omega.

[17] Murugan K., Rao T. N., Gandhi A. S., Murty B. S. (2010). Effect of Aggregation
of Methylene Blue Dye on TiO_2_ Surface in Self-Cleaning
Studies. Catal. Commun..

[18] Kumar R., Kumar A., Mondal P. S. (2022). Study of
UV-Assisted Photocatalytic
Degradation of Organic Dye. Mater. Today Proc..

[19] Laftani Y., Chatib B., Boussaoud A., El Makhfouk M., Hachkar M., Khayar M. (2019). Optimization of Diazo
Dye Disappearance
by the UV/H_2_O_2_ Process Using the Box–Behnken
Design. Water Sci. Technol..

[20] Ding X., Gutierrez L., Croue J.-P., Li M., Wang L., Wang Y. (2020). Hydroxyl and
Sulfate Radical-Based Oxidation of RhB Dye in UV/H_2_O_2_ and UV/Persulfate Systems: Kinetics, Mechanisms,
and Comparison. Chemosphere.

[21] Khataee A. R., Habibi B. (2010). Photochemical Oxidative
Decolorization of C.I. Basic
Red 46 by UV/H_2_O_2_ Process: Optimization Using
Response Surface Methodology and Kinetic Modeling. Desalin. Water Treat..

[22] Claus H. (2021). Ozone Generation
by Ultraviolet Lamps. Photochem. Photobiol..

[23] Lee D.-E., Kim M.-K., Danish M., Jo W.-K. (2023). State-of-the-Art
Review on Photocatalysis for Efficient Wastewater Treatment: Attractive
Approach in Photocatalyst Design and Parameters Affecting the Photocatalytic
Degradation. Catal. Commun..

[24] Chakravorty A., Roy S. (2024). A Review of Photocatalysis:
Basic Principles, Processes, and Materials. Sustainable Chem. Environ..

[25] Zhao D., Yang Y., Binas V., Shen S. (2025). Interface
Engineering
of Z-Scheme Heterojunction for Photocatalytic Water Splitting. Fundam. Res..

[26] von
Ackeret V. (1930). Experimentalle und theoretische Untersuchungen über
Hohlraumbildung (Kavitation) im Wasser. Technol.
Mech. Thermodyn..

[27] Yeneneh A. M., Balushi K. A., Jafary T., Al Marshudi A. S. (2024). Hydrodynamic
Cavitation and Advanced Oxidation for Enhanced Degradation of Persistent
Organic Pollutants: A Review. Sustainability.

[28] Mukherjee A., Mullick A., Teja R., Vadthya P., Roy A., Moulik S. (2020). Performance and Energetic Analysis of Hydrodynamic
Cavitation and Potential Integration with Existing Advanced Oxidation
Processes: A Case Study for Real Life Greywater Treatment. Ultrason. Sonochem..

[29] Doltade S. B., Dastane G. G., Jadhav N. L., Pandit A. B., Pinjari D. V., Somkuwar N., Paswan R. (2019). Hydrodynamic
Cavitation as an Imperative
Technology for the Treatment of Petroleum Refinery Effluent. J. Water Process Eng..

[30] Patil V. V., Gogate P. R., Bhat A. P., Ghosh P. K. (2020). Treatment
of Laundry
Wastewater Containing Residual Surfactants Using Combined Approaches
Based on Ozone, Catalyst, and Cavitation. Sep.
Purif. Technol..

[31] Pawar S. K., Mahulkar A. V., Pandit A. B. (2017). Sonochemical
Effect
Induced by Hydrodynamic Cavitation: Comparison of Venturi/Orifice
Flow Geometries. AIChE J..

[32] Thanekar P., Gogate P. R. (2019). Combined Hydrodynamic Cavitation-Based Processes as
an Efficient Treatment Option for Real Industrial Effluent. Ultrason. Sonochem..

[33] Jain P., Bhandari V. M., Balapure K., Jena J., Ranade V. V., Killedar D. J. (2019). Hydrodynamic Cavitation
Using Vortex Diode: An Efficient
Approach for Elimination of Pathogenic Bacteria from Water. J. Environ. Manage..

[34] Sun X., Xuan X., Song Y., Jia X., Ji L., Zhao S., Yoon J. Y., Chen S., Liu J., Wang G. (2021). Experimental and Numerical Studies on Cavitation in an Advanced Rotational
Hydrodynamic Cavitation Reactor for Water Treatment. Ultrason. Sonochem..

[35] Kim H., Koo B., Sun X., Yoon J. Y. (2020). Investigation of Sludge Disintegration
Using Rotor–Stator Type Hydrodynamic Cavitation Reactor. Sep. Purif. Technol..

[36] Vilarroig J., Martínez R., Zuriaga-Agustí E., Torro S., Galián M., Chiva S. (2020). Design and Optimization
of a Semi-Industrial
Cavitation Device for Pretreatment of Anaerobic Digestion of Excess
Sludge and Pig Slurry. Water Environ. Res..

[37] Marsalek B., Zezulka S., Marsalkova E., Pochylý F., Rudolf P. (2020). Synergistic Effects of Trace Concentrations
of Hydrogen
Peroxide Used in a Novel Hydrodynamic Cavitation Device Allow for
Selective Removal of Cyanobacteria. Chem. Eng.
J..

[38] Maleki M., Talabazar F. R., Davoudian S. H., Dular M., Kosar A., Petrovsek M., Smid A., Zupanc M., Ghobani M. (2025). The Formation
of Hydroxyl Radicals during Hydrodynamic Cavitation in Microfluidic
Reactors Using Salicylic Acid Dosimetry. Chem.
Eng. J..

[39] Abdulaziz A. M. (2014). Performance
and Image Analysis of a Cavitating Process in a Small-Type Venturi. Exp. Therm. Fluid Sci..

[40] Kawano K., Oka Y. (2025). Hydroxyl Radical Formation
and Its Mechanism in Cavitation Bubble
Plasma-Treated Water: A Chemical Probe Study. Liquids.

[41] Ye Y.-F., Zhu Y., Lu N., Wang X., Su Z. (2021). Treatment of Rhodamine
B with Cavitation Technology: Comparison of Hydrodynamic Cavitation
with Ultrasonic Cavitation. RSC Adv..

[42] Zhang X., Hao C., Ma C., Shen Z., Guo J., Sun R. (2019). Sonocatalytic
Degradation of Rhodamine B in Aqueous Solution. Ultrason. Sonochem..

[43] Mancuso G., Langone M., Laezza M., Andreottola G. (2016). Decolourization
of Rhodamine B: A Swirling Jet-Induced Cavitation Combined with NaOCl. Ultrason. Sonochem..

[44] Kumar M. S., Sonowane S. H., Pandit A. B. (2017). Degradation
of Methylene Blue Dye
in Aqueous Solution Using Hydrodynamic Cavitation-Based Hybrid Advanced
Oxidation Processes. Chem. Eng. Process. –
Process Intensif..

[45] Sugha A., Bhatti M. S. (2022). Degradation of Methylene
Blue Dye by UV/H_2_O_2_ Advanced Oxidation Process:
Reaction Kinetics, Residual
H_2_O_2_ and Specific Energy Consumption Evaluation. Desalin. Water Treat..

[46] Bagal M. V., Rajan V. B., Shinde S. S., Banerjee B. S., Gole V. L., Mohod A. V. (2024). Degradation of Azo Dyes Using Hydrodynamic Cavitation
and External Oxidants. Adv. Environ. Technol..

[47] Sarvothaman V. P., Velisoju V. K., Subburaj J., Panithasan M. S., Kulkarni S. R., Castaño P., Turner J., Guida P., Roberts W. L., Nagarajan S. (2023). Is Cavitation
a Truly Sensible Choice
for Intensifying Photocatalytic Oxidation Processes? Implications
on Phenol Degradation Using ZnO Photocatalysts. Ultrason. Sonochem..

[48] Mahdavi R., Talesh S. S. A. (2021). Enhanced Selective
Photocatalytic and Sonocatalytic
Degradation in Mixed Dye Aqueous Solution by ZnO/GO Nanocomposites:
Response Surface Methodology. Mater. Chem. Phys..

[49] Fedorov K., Dinesh K., Sun X., Soltani R. D. C., Wang Z., Sonawane S., Boczkaj G. (2022). Synergistic
Effects of Hybrid Advanced
Oxidation Processes (AOPs) Based on Hydrodynamic Cavitation Phenomenon
– A Review. Chem. Eng. J..

[50] Gągol M., Cako E., Fedorov K., Soltani R. D. C., Przyjazny A., Boczkaj G. (2020). Hydrodynamic Cavitation-Based Advanced
Oxidation Processes:
Studies on Specific Effects of Inorganic Acids on the Degradation
Effectiveness of Organic Pollutants. J. Mol.
Liq..

[51] Hwang T.-M., Nam S.-H., Lee J., Koo J.-W., Kim E., Kwon M. (2020). Hydroxyl Radical Scavenging
Factor Measurement Using a Fluorescence
Excitation–Emission Matrix and Parallel Factor Analysis in
Ultraviolet Advanced Oxidation Processes. Chemosphere.

[52] Cheng X., Liang L., Ye J., Li N., Yan B., Chen G. (2023). Influence and Mechanism of Water
Matrices on H_2_O_2_-Based Fenton-Like Oxidation
Processes: A Review. Sci. Total Environ..

[53] Bolton J. R., Bircher K. G., Tumas W., Tolman C. A. (2001). Figures of Merit
for the Technical Development and Application of Advanced Oxidation
Technologies for Both Electric- and Solar-Driven Systems. Pure Appl. Chem..

[54] Brunhart M., Soteriou C., Gavaises M., Karathanassis I., Koukouvinis P., Jahangir S., Poelma C. (2020). Investigation of Cavitation
and Vapor Shedding Mechanisms in a Venturi Nozzle. Phys. Fluids.

[55] Rosas, J. ; Naude, J. ; Méndez, F. ; Mayen, R. ; Navarrete, M. In Detachment of Cavitating Rings in a Long-Throat Venturi Tube; Proceedings of the 11th International Symposium on cavitation (CAV 2021); Daejon, Korea, Paper P00041;122–125. CAV http:www.cav2021.org.

[56] Navarrete M., Naude J., Méndez F., Godínez F. A. (2015). Dynamics
and Acoustics of a Cavitating Venturi Flow Using Homogeneous Air–Propylene
Glycol Mixture. J. Phys. Conf. Ser..

[57] Pelz P. F., Keil T., Grob T. F. (2017). The Transition
from Sheet to Cloud
Cavitation. J. Fluid Mech..

[58] van Terwisga, T. J. C. ; Hoyt, J. G., III ; Zangeneh, M. ; Choi, G.-I. ; Oloffson, N. ; Ranocchia, D. ; Sadovnikov, D. In The Specialist Committee on Water Quality and Cavitation; Proceedings of the 23rd International Towing Tank Conference (ITTC 2002), Vol. II de Bernardis, E. , Ed.; ITTC pp 459–491.

[59] Toyran E., Zupanc M., Petrovšek M., Dular M. (2026). The Importance of (Un)­Dissolved
Gases on Early-Stage Cavitation Dynamics within an Acoustic Field. Ultrason. Sonochem..

[60] Tomov P., Khelladi S., Ravelet F., Sarraf C., Bakir F., Vertenoeuil P. (2016). Experimental
study of aerated cavitation in a horizontal
venturi nozzle. Exp. Ther. Fluid Sci..

[61] Liao Z. M., Zhang H. Z., Zhou Y. B., Xu J., Zhang J. M., Yu D. P. (2008). Surface Effects on Photoluminescence
of Single ZnO Nanowires. Phys. Lett. A.

[62] Gurylev V., Perng T. P. (2021). Defect Engineering
of ZnO: Review on Oxygen and Zinc
Vacancies. J. Eur. Ceram. Soc..

[63] Modi S., Kumar V., Gacem A., Ali I. H., Dave D. (2022). Recent and Emerging
Trends in Remediation of Methylene Blue Dye from
Wastewater Using Zinc Oxide Nanoparticles. Water.

[64] Modi S., Kumar Yadav V., Amari A., Alyami A. Y., Gacem A., Harharah H. N., Fulekar M. H. (2023). Photocatalytic Degradation of Methylene
Blue Dye from Wastewater Using Doped Zinc Oxide Nanoparticles. Water.

[65] Ding Y., Niu L., Chen Y., Wang M. H. (2023). Study on the Defect Structure of
Carbon-Doped ZnO Materials. Cryst. Res. Technol..

[66] Guo H. L., Zhu Q., Wu X. L., Jiang Y. F., Xie X., Xu A. W. (2015). Oxygen-Deficient
ZnO_1–x_ Nanosheets with High Visible-Light Photocatalytic
Activity. Nanoscale.

[67] Kulis-Kapuscinska A., Kwoka M., Borysiewicz M. A., Wojciechowski T., Licciardello N., Sgarzi M., Cuniberti G. (2023). Photocatalytic
Degradation of Methylene Blue at Nanostructured ZnO Thin Films. Nanotechnology.

[68] Islam Md. M., Aidid A. R., Moshshin J. N., Mondal H., Ganguli S., Chakraborty A. K. (2025). A Critical Review on Textile Dye-Containing
Wastewater:
Ecotoxicity, Health Risk, and Remediation Strategies for Environmental
Safety. Cleaner Chem. Eng..

[69] Sievers, M. Advanced Oxidation Processes. In Treatise on Water Science; Wilderer, P. , Ed.; Elsevier, 2011; Chapter 4.13; pp 377–408 10.1016/B978-0-444-531199-5.00093-2.

[70] Gostisa J., Sirok B., Repinc S. K., Levstek M., Strazar M., Bizjan B., Zupanc M. (2021). Performance
Evaluation of a Novel-Scale
Pinned Disc Rotating Generator of Hydrodynamic Cavitation. Ultrason. Sonochem..

[71] Gagol M., Przyjazny A., Boczkaj G. (2018). Wastewater Treatment by Means of
Advanced Oxidation Process Based on Cavitation—A Review. Chem. Eng. J..

[72] Nopel J.-A., Ayela F. (2023). Experimental evidences of radicals
production by hydrodynamic cavitation:
short review. C. R. Chim..

[73] De-Nasri S. J., Sarvothaman V. P., Nagarajan S., Manesiotis P., Robertson P. K. J., Ranade V. V. (2022). Quantifying OH radical generation
in hydrodynamic cavitation via coumarin dosimetry: Influence of operating
parameters and cavitation devices. Ultrason.
Sonochem..

[74] Mohamadpour F., Amani A. M. (2024). Photocatalytic systems: reactions, mechanism, and applications. RSC Adv..

